# The New Registration Act

**Published:** 1836-10

**Authors:** 


### THE NEW REGISTRATION ACT.

IT is most gratifying to every friend of humanity to observe the progressive advances now daily making in all the peaceful arts, and the increased attention of all free governments towards what may be termed social and scientific legislation. A striking instance of this we have in the Registration Bill passed by our legislature during the last Session, which goes a great way towards supplying some of the most important desiderata in medical statistics. We are by no means sure that the machinery which has been adopted for the purpose of carrying into effect the provisions of the act, is the best that might have been chosen, and we have apprehensions, accordingly, respecting its ready and effective working; but there can be no doubt that a great boon has been conferred on society by it; and we do not fear to see remedied any practical defects that it may now involve. The great thing was to get the legislature to make a commencement.

The act is entitled " An Act for registering Births, Deaths, and Marriages in England," and it effectually provides for the accurate registration of all these events, without regard to the station, religion, or any other condition of the individual.

By this act, a *Superintendent Registrar* is to be attached to every parochial union established by the Poor-Law Amendment Act, and a *Registrar* to each district into which such union may be divided. The registrars are to keep a record of the births, deaths, and marriages occurring in their respective districts, which they are to transmit quarterly to the superintendant registrar of the union, who is to transmit them to the office of the *Registrar General* in London, also quarterly. An abstract of all these registrations throughout England is to be laid annually before parliament, and will become, as a matter of course, accessible as a public document to every one. Effectual provisions are made for rendering the registration imperative in all cases.

Of all the particulars included in this act, there is none so interesting to the medical profession as that which enjoins the *cause of death* to be registered in each case; and, if due care is taken to have this part of the duty properly performed, it cannot be doubted but that the most valuable information respecting the laws of health and disease will eventually be therethrough obtained, with the necessary result of ameliorating the health of the community at large, and extending the mean term of human life in this country.

The following are the particulars enjoined to be recorded in each case of birth, death, and marriage, respectively:

| Births. | Deaths. | Marriages. |
|---|---|---|
| 1. No. in the register. | 1. No. in the register. | 1. No. in the register. |
| 2. When born. | 2. When died. | 2. When married. |
| 3. Name, if any. | 3. Name and surname. | 3. Name and surname. |
| 4. Sex. | 4. Sex. | 4. Age. |
| 5. Name and surname of father. | 5. Age. | 5. Condition. |
| 6. Name and maiden surname of mother. | 6. Rank or profession. | 6. Rank or profession. |
| 7. Rank or profession of father. | 7. Cause of death. | 7. Residence. |
| 8. Signature, description, and residence of informant. | 8. Signature, description, and residence of informant. | 8. Father's name and surname. |
| 9. When registered. | 9. When registered. | 9. Rank or profession of father. |
| 10. Baptismal name, if added after registration of birth. | | |

